# Genome-wide identification and characterization of InDels and SNPs in *Glycine max* and *Glycine soja* for contrasting seed permeability traits

**DOI:** 10.1186/s12870-018-1341-2

**Published:** 2018-07-09

**Authors:** G. Ramakrishna, Parampreet Kaur, Deepti Nigam, Pavan K. Chaduvula, Sangita Yadav, Akshay Talukdar, Nagendra Kumar Singh, Kishor Gaikwad

**Affiliations:** 1 ICAR- National Research Centre on Plant Biotechnology, Pusa Campus, New Delhi, 110012 India; 2 ICAR- IARI, Division of Seed Science and Technology, Pusa Campus, New Delhi, 110012 India; 3 ICAR- IARI, Division of Genetics, Pusa Campus, New Delhi, India

**Keywords:** Seed permeability, NextGen sequencing, Soybean, SNPs, InDels

## Abstract

**Background:**

Water permeability governed by seed coat is a major facet of seed crops, especially soybean, whose seeds lack physiological dormancy and experience rapid deterioration in seed viability under prolonged storage. Moreover, the physiological and chemical characteristics of soybean seeds are known to vary with seed coat color. Thus, to underpin the genes controlling water permeability in soybean seeds, we carried out an in-depth characterization of the associated genomic variation.

**Results:**

In the present study, we have analyzed genomic variation between cultivated soybean and its wild progenitor with implications on seed permeability, a trait related to seed storability. Whole genome resequencing of *G.max* and *G. soja*, identified SNPs and InDels which were further characterized on the basis of their genomic location and impact on gene expression. Chromosomal density distribution of the variation was assessed across the genome and genes carrying SNPs and InDels were characterized into different metabolic pathways. Seed hardiness is a complex trait that is affected by the allelic constitution of a genetic locus as well as by a tricky web of plant hormone interactions. Seven genes that hold a probable role in the determination of seed permeability were selected and their expression differences at different stages of water imbibition were analyzed. Variant interaction network derived 205 downstream interacting partners of 7 genes confirmed their role in seed related traits. Interestingly, genes encoding for Type I- Inositol polyphosphate 5 phosphatase1 and E3 Ubiquitin ligase could differentiate parental genotypes, revealed protein conformational deformations and were found to segregate among RILs in coherence with their permeability scores. The 2 identified genes, thus showed a preliminary association with the desirable permeability characteristics.

**Conclusion:**

In the light of above outcomes, 2 genes were identified that revealed preliminary, but a relevant association with soybean seed permeability trait and hence could serve as a primary material for understanding the molecular pathways controlling seed permeability traits in soybean.

**Electronic supplementary material:**

The online version of this article (10.1186/s12870-018-1341-2) contains supplementary material, which is available to authorized users.

## Background

Soybean is cultivated for two economically and nutritionally important compounds, i.e., protein and oil, which together constitute approximately 56% of its dry seed weight. Wild (*G. soja*) and cultivated soybean (*G. max*) differ in various morphological and physiological characteristics. Large seeds with variable seed coat colors are characteristic of cultivated varieties and referred to as permeable seeds, whereas wild species possess small, coarse black and hard seeds that display water impermeability. Wild and cultivated soybean differ in the extent of hardseededness, though considerable variation exists in the later for water permeability [1–3]. Hardseededness has a multitude of effects that raises both biological and economic concerns as it enables perseverance of seed stocks for several years [4] by providing resistance and protection from seed spoilage and seed pathogens but poses a major problem for seed germination that is required to generate high crop yields [4, 5]. Thus, moderately impermeable seed cultivars are desirable as they can maintain their post-harvest viability and quality.

Both environmental and genetic factors contribute towards soybean seed permeability. Morphologically, hardseededness is related to the absence of cracks in seed coat [6]. Complex multilayer seed coat and its chemical constituents are crucial determinants of a water barrier system of the seed coat, and presence of phenolic compounds has also been associated with seed coat impermeability [2, 7]. Numerous studies have associated insoluble lipid polyesters (cutin and cutin like depositions) in seed coat of Arabidopsis [8], brassica [9] and soybean [10] with seed impermeability. In Arabidopsis, mutations in genes such as LAC15 [11], acyl-CoA:glycerol-3-phosphate acyltransferase, suberin biosynthetic gene GPAT5 [12] etc., has led to higher permeability than the wild-type seeds. Phytohormones such as abscisic acid (ABA) also play an important role in the regulation of seed germination [13–15] and triggers ABI5 accumulation and phosphorylation to repress germination [16]. In Arabidopsis [17] and Medicago [18], ABI5 is identified as a prominent regulator of seed maturation and longevity. To understand the genetic basis of seed hardiness, different studies have reported the presence of a common QTL on an overlapping region of soybean chromosome 2 [19–22]. Sun et al. has delimited this QTL to a 22Kb region containing 2 genes, of which SNP (C > T) in the 8th exon of Glyma02g43700.1, designated as *GmHs1–1* and encoding for Calcineurin like metallophosphoesterase transmembrane protein in malphigian layers of seed coat, could effectively distinguish between parental genotypes and 8 additional *G. soja* accessions [23]. Another locus positioned closely to GmHs1–1 is qHS1QTL, which encodes for endo-1,4-ßglucanases and results in accumulation of ß1- 4glucan derivatives that reinforces hardseededness in soybean [24].

In the present study, we have attempted to identify variation (SNPs and InDels) between genomes of *G. max* and *G. soja* in relation to their seed permeability characteristics. SNPs and InDels were categorized into different metabolic pathways and genes were selected from pathways that could influence seed permeability, followed by their validation through real-time expression studies. Using *In-silico* approaches, various downstream analysis were conducted for candidate genes to reveal an association between their structure and permeability. Further, we have shown that genomic variation in the selected genes could be further developed into markers to distinguish permeable and impermeable parental genotypes and their RILs in coherence with their permeability scores. Although the numbers of SSR and RFLP markers are known to be associated with seed permeability and hardseededness, this is the primary but comprehensive report on identification and association of genome-wide SNP and InDels with seed permeability.

## Results

### Resequencing, mapping, and assembly of *G. max* and *G. soja*

**Fig. 1:**
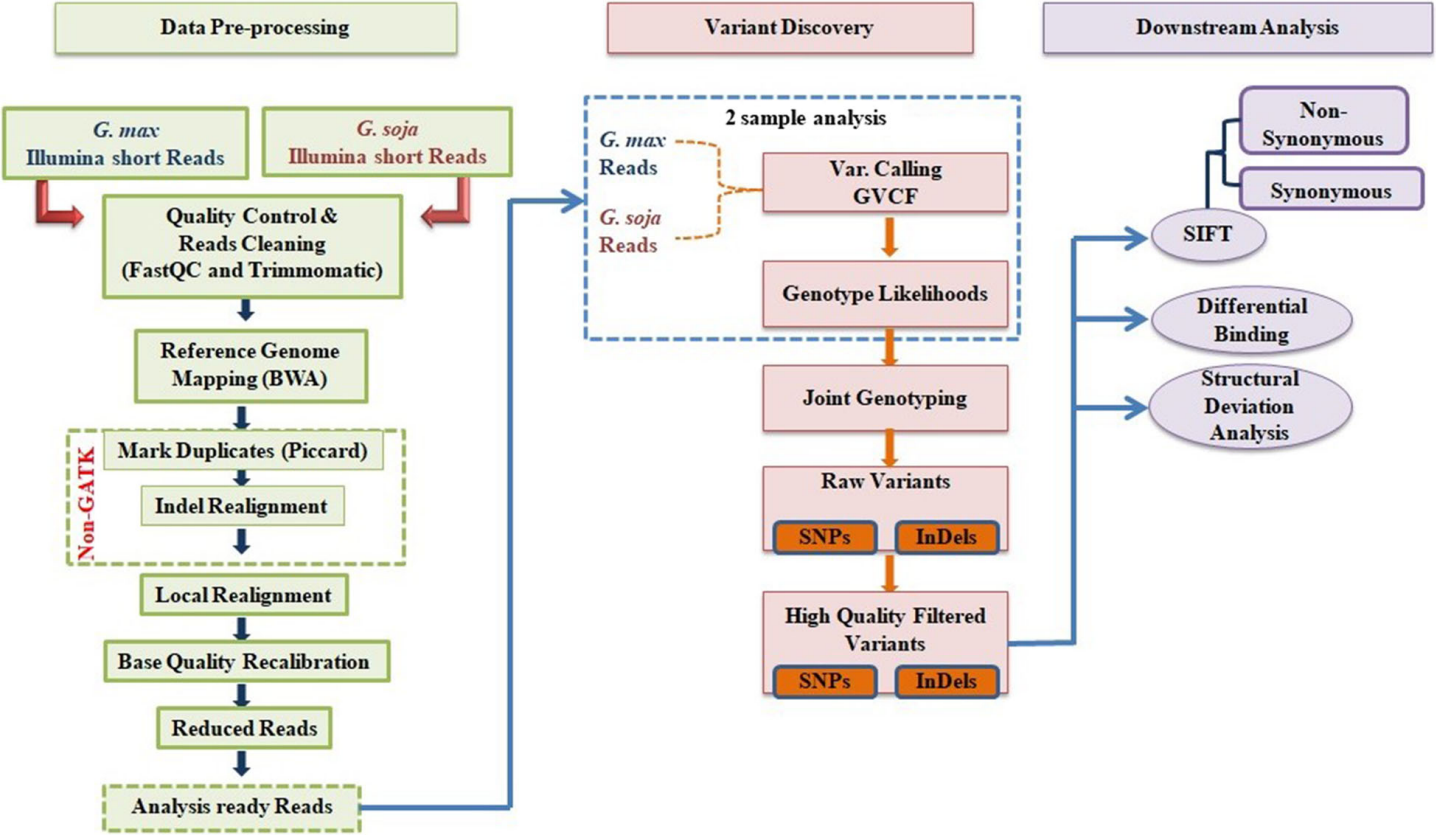
Workflow diagram for SNPs and InDels identification in *G. max (Glycine max) and G. soja (Glycine soja)*. Workflow is divided into three categories: Data quality pre-processing, Discovery of SNPs and InDels and Downstream Analysis of High-Quality Filtered SNPs and InDels

Workflow diagram of the analysis done is shown in Fig. 1. A total of 118.8 million and 119.8 million potentially paired-end reads of *G. max* and *G. soja* were obtained respectively. Sequence information has been deposited in the BioProject database of NCBI, under ID PRJNA383915. A total of 117.11 M (98.57%) and 113.66 M (94.87%) high quality filtered reads of *G. max* and *G. soja* were successfully mapped to the reference genome (*G. max* var. Williams 82) with a coverage of 12.56 and 12.32 X, respectively (Additional file 1: Table S1).

### Identification and chromosomal distribution of SNPs and InDels

A total of 77,339 and 215932 SNPs as well as 451,522 and 697,295 InDels were identified in *G. max* and *G. soja*, respectively, upon comparison with the reference genome after filtering. The average density of SNPs and InDels in *G. max* was observed to be about 79.04/Mb and 461.48 /Mb and 291.01 SNPs/Mb and 712.67 InDels/Mb in *G. soja*. In total, 10,873 SNPs and 80,078 InDels were found to be common to both species. A total of 40,130 SNPs and 381,644 InDels of *G. max* could be mapped onto 7903 and 32,116 genes, respectively, while in *G. soja* 154,611 SNPs and 595,433 InDels were mapped onto 29,823 and 51,310 genes, respectively. A total of 1760 genes in *G. max* (Additional file 2) and 29,530 genes in *G. soja* (Additional files 3 and 4) were found to possess both SNPs and InDels.

Numbers of transition (Ts) and transversion (Tv) SNPs identified in *G. max* have shown Ts/Tv ratio of 1.814 and 1.859 in. *G. soja* A/T and G/T represented most frequent transversion while frequencies of C/T and A/G transitions were similar in both species. Ts/Tv ratio of 2.81, 2.65 and 3.41 was observed at first, second and third codon position, respectively. Deletions and insertions of length observed in *G. max* ranged from 1 to 90 bp and 1 to 75 bp, respectively, while a range of 1 to 110 bp and 1 to 80 bp, respectively was observed for *G. soja*.

**Fig. 2:**
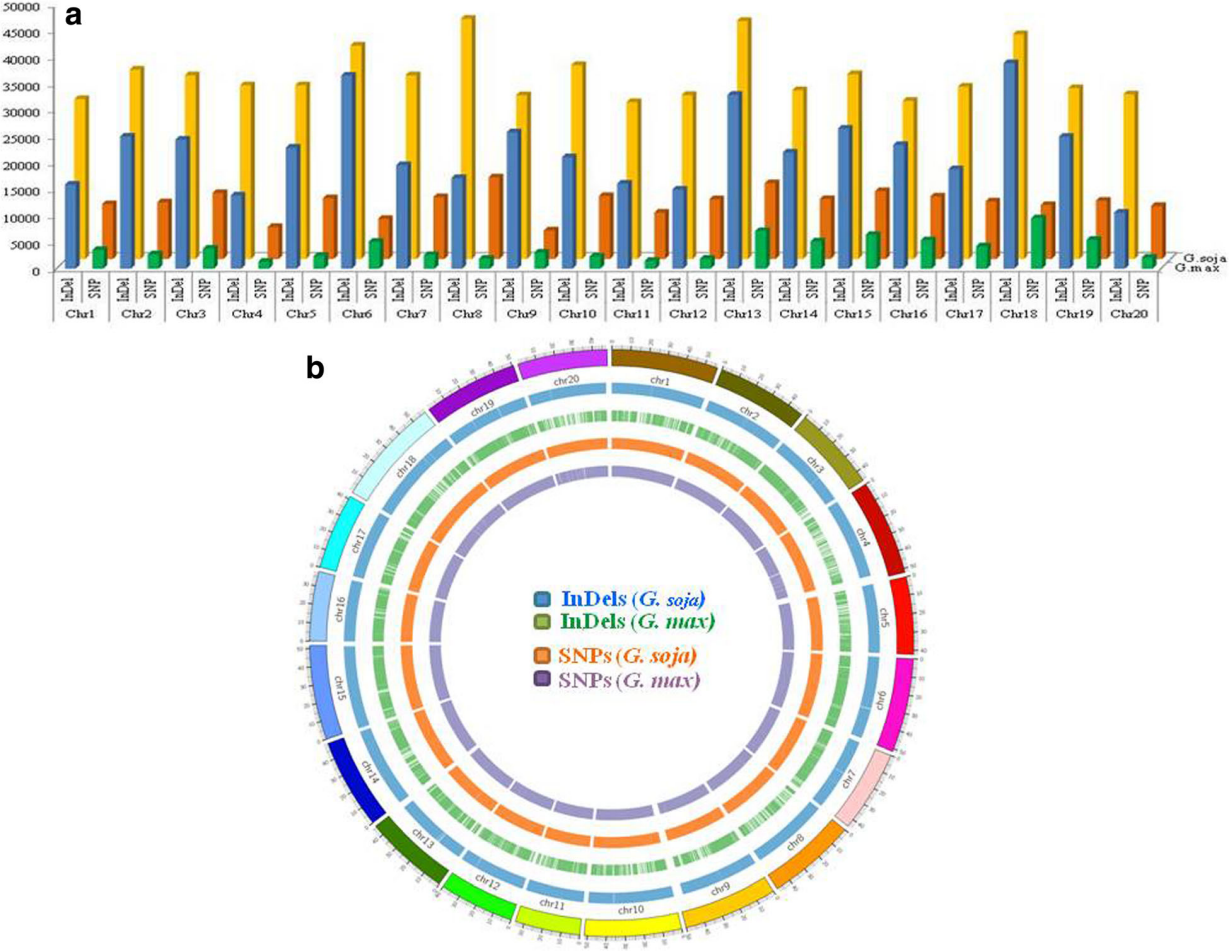
Frequency (**a**) and density distribution (**b**) of SNPs and InDels detected across the 20 chromosomes of *G. max* and *G. soja*. The number of SNPs and InDels detected are illustrated as bar graphs in different colors (Y-axis represents the frequency of variation and the X-axis represents the type of variation and chromosome numbers) while the density distribution of identified variation is reflected as a Circos plot (1 Mb window size)

In both species, non-uniform distribution of SNP and InDels across 20 chromosomes was observed (Fig. 2a). Chromosome 18 is the largest chromosome and possessed the highest number of InDels and SNPs in *G. max* i.e., 670.47 InDels/Mb and 165.18 SNPs/Mb. While the least number of InDels (221.77 InDels/Mb) and SNPs (24.66 SNPs/Mb) were mapped on chromosome 20 and chromosome 4 of *G. max*, respectively. Chromosome 8 of *G. soja*, possessed a maximum number of InDels (951.23 InDels/Mb) and SNPs (322.86 SNP/Mb) across its genome. The least number of SNPs and InDels were mapped onto chromosome 9 (108.02 SNPs/Mb) and chromosome 11 (855.30 InDels/Mb) of *G. soja*, respectively. Further, for both species, most dense distribution of variation towards the chromosome ends was observed (Fig. 2b). Less InDels were detected with increasing length while different InDels of the same length shared similar abundance in both species.

### Classification and categorization of SNPs and InDels

SNPs and InDels were further categorized into various groups (Table 1). InDels and SNPs were more abundantly observed in upstream and downstream regulatory regions (URR and DRR, respectively) of genes. A total of 1876 InDels resulted in translation frame-shifts, while 1227 InDels caused deletion/insertion of amino acids in *G. max* whereas in *G. soja* the number ranged from 2287 and 1892 InDels, respectively. A total of 60 and 219 SNPs in *G. max* and *G. soja*, respectively, were found to affect essential splice donor or acceptor sites. Impact of variation in both species collectively was categorized as: low impact (0.22%), modifier (99.08%), moderate (0.35%), and high impact variation (0.34%). Overall, a high frequency of SNPs and InDels was observed to influence the gene function in *G. soja* relative to *G. max*. In both *G. max* and *G. soja*, low impact SNPs were higher in number compared to that of high impact SNPs, but a reverse trend was observed for low and high impact InDels. Collectively, 125 high impact SNPs and 4594 InDels were identified in both species which affected 1459 and 1839 genes in *G. max* and *G. soja,* respectively*.* Cumulatively, in *G. max* and *G. soja*, 45 and 83 high impact SNPs and InDels, respectively, were identified to result in a gain of stop codon while 22 and 40 high impact SNPs and InDels, respectively accounted for the loss of stop codon in a transcript. Approximately, 90 and 75% of the low impact SNPs represented synonymous SNPs in *G. max* and *G. soja*, respectively, wherein an amino acid encoded by a gene remained unaltered thus imparting low impact on gene functionality. Modifier SNPs were mostly represented in upstream and downstream gene variants in both *G. max* and *G. soja*. Approximately, 82% of the high impact InDels resulted in frameshift variation in *G. max* and *G. soja* while the low impact InDels were present in intron regions and splice site regions. Further, 70 and 148 SNPs in *G. max* and *G. soja*, respectively, were found to possess SIFT score ≤ − 2.5 and were termed as deleterious. Among non-synonymous variants, 69 and 143 SNPs in *G. max* and *G. soja,* respectively, were deleterious.

**Table 1:** Categorization of SNPs and InDels in *G. max* and *G. soja* (URR: Upstream regulatory regions, DRR: Downstream regulatory regions, CDS: Coding sequences)

Variations	Type of Variation		Frequency in *G. max*	Frequency in *G. soja*
SNPs	Gene Based	URR	11,508	73,384
		DRR	9546	60,406
		CDS	4386	20,831
	Intergenic		925	31,936
	Non-synonymous		380	903
InDels	Gene Based	URR	153,820	265,741
		DRR	133,212	228,455
		CDS	62,335	101,156
	Intergenic		65,090	101,823
	Non-synonymous		1077	2013

Further, non-synonymous SNPs and InDels were mapped uniquely onto 76 pathways in *G. max* and 102 pathways in *G. soja*. Interestingly, 9 and 35 pathways were unique to *G. max* and *G. soja*, respectively, whereas 67 pathways were found to be common between them. This classification identified a diverse array of pathways ranging from cellular metabolism, transcriptional regulators for genes functioning under stress conditions as well as defense pathways (Additional file 5).

### Analysis of differential binding motif and variant interaction network (VIN) reveals new players governing seed permeability differences

**Fig. 3:**
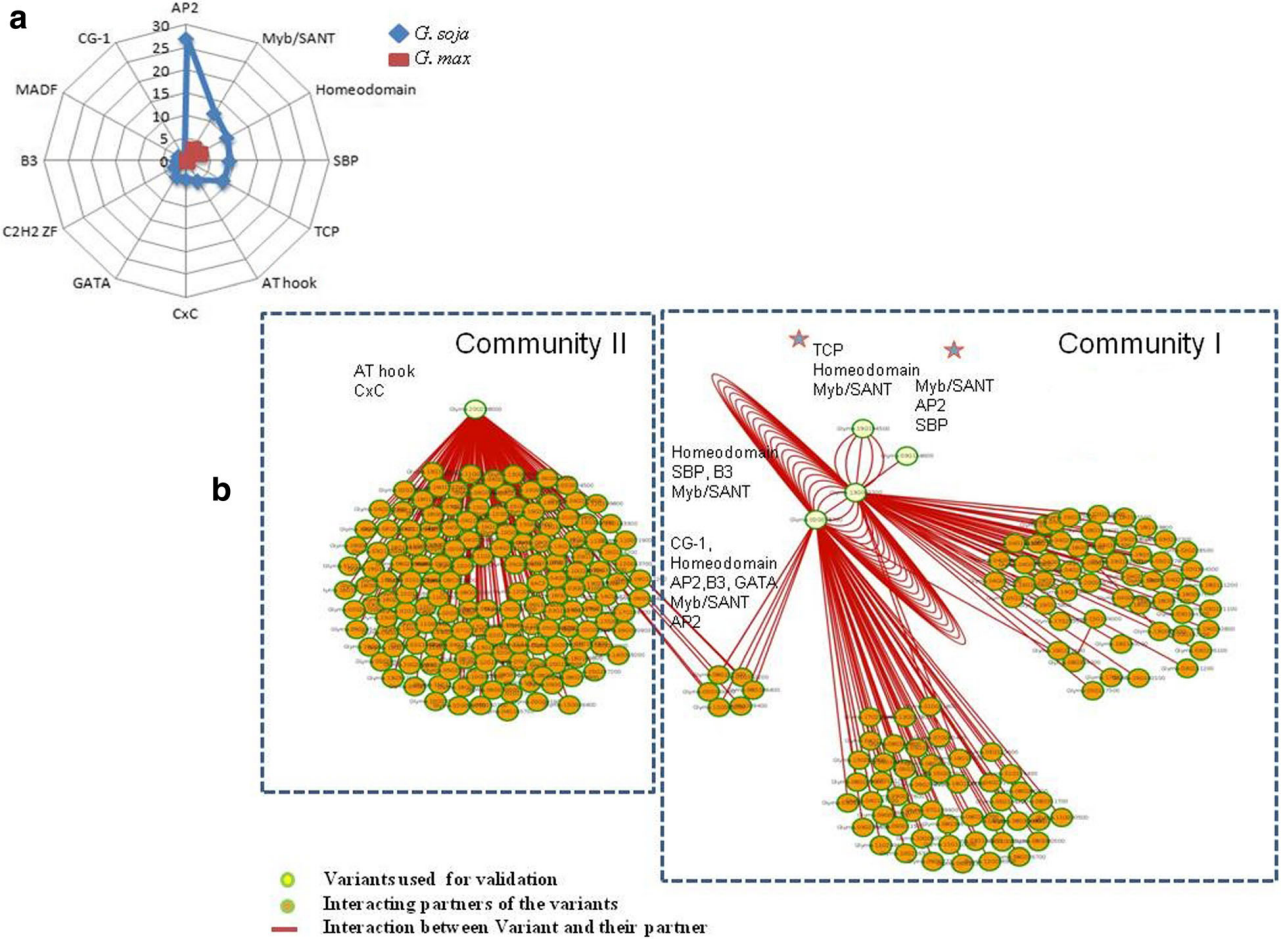
Frequency of differential binding motifs identified at variation position of 7 genes between *G. max* and *G. soja* (Numeric indicating motif frequency) (**a**); Variant Interaction Network (VIN) representing the interacting partners for seven genes in two major communities (**b**)

Seven genes carrying non-synonymous variants having deleterious effects on gene function were found to be involved in seed permeability related metabolic pathways (Table 2). The variable region of these genes accounted for differential binding of 13 transcription factor (TF) motifs between *G. soja* and *G. max* (Fig. 3a). The higher occurrence of motifs for AP2, Myb/SANT, Homeodomain, SBP, TCP, AT hook, CxC, GATA, C2H2 ZF, B3, MADF, and CG-1 were observed in *G. soja* in comparison to *G. max*. Interestingly, except Myb/SANT and homeodomain motif, other 10 motifs were observed as unique to the *G. soja*.

**Table 2:** Features of genes involved in seed coat permeability selected for further analysis (Chr. No. - Chromosome Number; bp – base pairs)

Gene (Glyma Ids)	Gene length (bp)	Variation position (Chr No.: start position coordinates)	Variation identified (*G.max* / *G.soja*)	Variation effect (*G. max/G. soja* amino acid change) on protein	Impact of variability
Anthranilate n-hydroxy cinnamoyl/benzoyl transferase (Glyma17g16330)	4249	17:12977991	−−−/AAA	(−/K)	Moderate
Chalcone flavone isomerase (Glyma03g31100)	6050	3:38990976	−−−−−−−−−/ACGGCCACG	(−/VAV)	Moderate
Abscisic Acid Insensitive 5 (Glyma19g37910)	5266	19:45001036	−−−/GGT	(−/G)	Moderate
PhospholipaseD (Glyma20g38200)	10,132	20:45855223	−−−/TTG	(−/L)	Moderate
TypeI- Inositol polyphasphate 5 phosphotase1 (Glyma02g08620)	7867	2:6711434	−−−−−−−−−−−−/GGACAACAGGCA	(−/DNRC)	Moderate
E3 ubiquitin ligase (Glyma10g43120)	4938	10:49932855	−−−−−−−−−−/TATAGATATA	Stop codon gain, Protein truncation (P/L*IYX)	High
Glycosyl transferase (Glyma13g05441)	5969	13:5763533	−−/TA (Insertion is present upstream to gene)	–	Moderate

To further explore the possible effect of variation identified in the context of their role in mediating differential TF’s binding events, interaction network of 7 selected genes (guide genes) was constructed that identified 205 interacting partners distributed in 2 network communities (Fig. 3b). The community I was regulated by 1 guide gene, i.e., Phospholipase D (Glyma20g38200) and was enriched for TFs with AT-hook and CxC motif, while community II was regulated by 4 guide genes viz. Glycosyltransferases (Glyma13g05441), Chalcone flavone isomerase (Glyma03g31100), ABI5 (Glyma19g37910) and Type I/Type I/InsP 5-ptase (Glyma02g08620) and these hub genes were enriched with differential binding motifs for AP2, Myb/SANT, Homeodomain, SBP, TCP, B3, CG-1, AP2, B3 (as complex) and GATA TFs, wherein AP2, Myb/SANT, Homeodomain, SBP were represented in at least 2 guide genes. Further, these 205 downstream interacting partners were annotated and classified into biological processes, molecular functions and cellular components (Additional file 6: Figure S1, Additional file 7: Figure S2, Additional file 8: Figure S3; Additional file 9), with the majority of annotations specific to seed related traits.

### DNA polymorphism and real-time expression studies

**Fig. 4:**
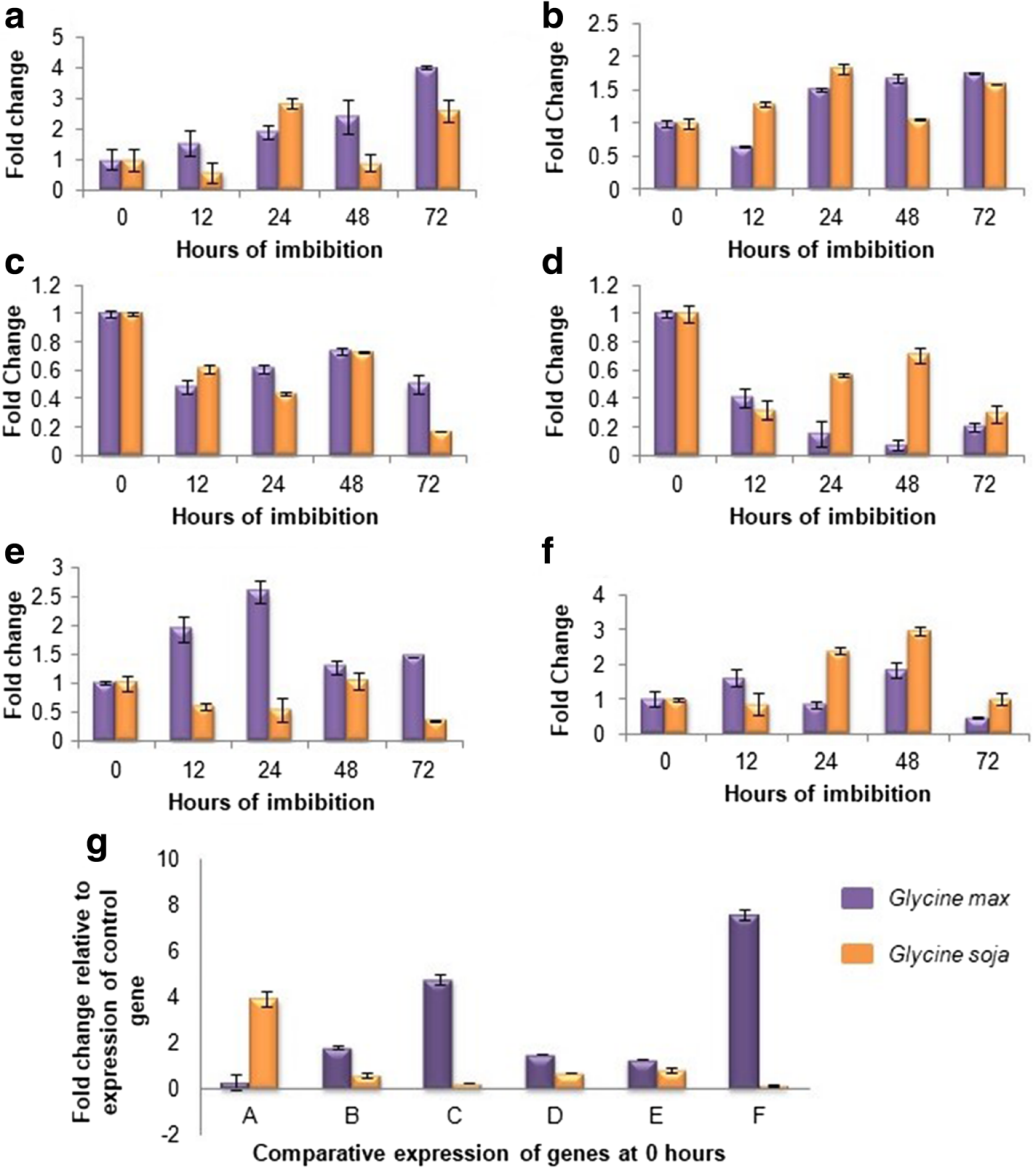
Real-time expression studies of 6 genes in *G. max* and *G. soja* at 0, 12, 24, 48 and 72 h of imbibition along with computed standard errors (**a**: Glycosyl transferases; **b**: Phospholipase D; **c**: Anthranilate n-hydroxy cinnamoyl/benzoyl transferase; **d**: Abscisic acid insensitive 5, **e**: Chalcone flavone isomerase; **f**: E3 Ubiquitin ligase; **g**: Comparative expression of genes A-F at 0 h in *G. max* and *G. soja* normalized to the expression data of 18 s rRNA gene)

DNA polymorphism observed between parental genotypes for all genes was in accordance to in silico predictions (Additional file 10: Figure S4). Except for gene Type I Inositol Polyphosphate 5 Phosphatase1 (Type I/InsP 5-ptase), for which no efficient real-time primers could be designed, another 6 genes were analyzed for differences in transcript abundance at 5 different time intervals of water imbibition of the seed (Fig. 4).

The number of transcripts of Glycosyltransferases were found to increase during imbibition, with 2.5 fold increase at 72 h in comparison to control whereas *G. soja* transcripts were relatively less abundant, though a transient increase was observed in comparison to *G. max* at 24 h of imbibition. A slow increase in transcript level of Phospholipase D occurred in *G. max* in contrast to *G. soja*. Maximum number of transcripts were present at 24 h of imbibition in *G. soja* followed by a decline thereafter. A gradual decline in the transcript level of Anthranilate n-hydroxy cinnamoyl/benzoyl transferase (HCBT) was observed in *G. soja* with a transient increase at 48 h of imbibition. In *G. max*, HCBT transcripts were present at low levels during imbibition in comparison to control seeds, though an increase in transcript number was observed from 12 to 48 h of imbibition followed by a dip thereafter. Transcripts of Abscisic acid insensitive 5 (ABI5) were present in lower amounts in both *G. max* and *G. soja* during imbibition in comparison to that of control seeds. An antagonistic pattern of ABI5 transcript abundance was observed between *G. max* and *G. soja.* A 2 fold increase observed in transcript level of Chalcone flavone isomerase in *G. max* was in contrast to 2 fold reduction observed for transcripts in *G. soja* until 24 h of imbibition. This was followed by a decline in transcripts of *G. max* and increased expression in *G. soja* at 48 h of imbibition. The transcript level of E3 Ubiquitin ligase did not reveal any particular pattern of abundance, though the transcript level increased gradually from 24 to 48 h followed by a dip thereafter in *G. soja*, an antagonistic expression pattern was observed in *G. max* and *G. soja* with respect to each other.

### In silico characterization of effect of variation on amino acid composition and protein structure

**Fig. 5:**
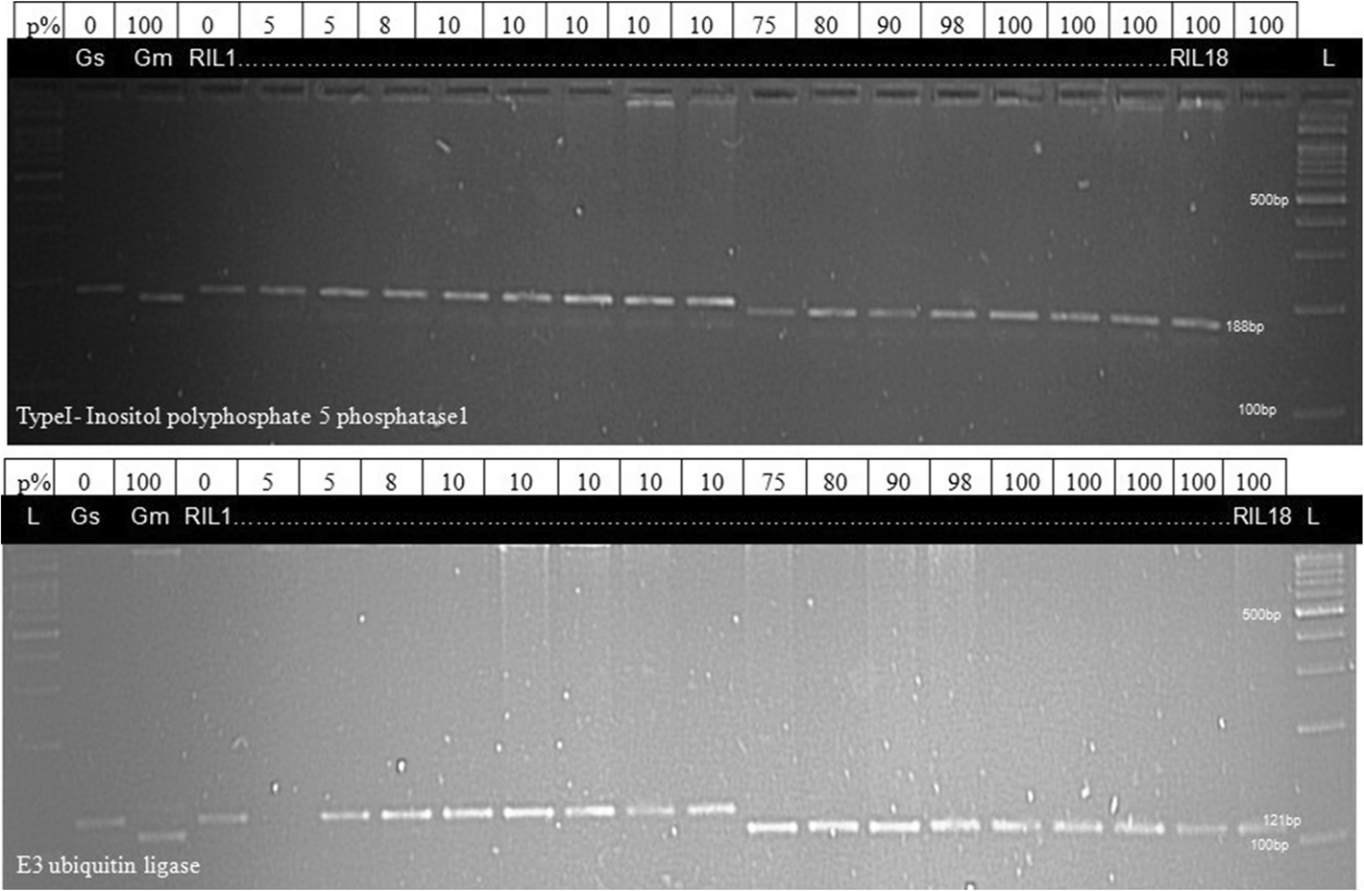
Segregation pattern observed for genes viz. TypeI- Inositol polyphosphate 5 phosphatase1 (top) and E3 ubiquitin ligase (bottom) among 18 RILs (water permeability scores are depicted in a row above the gel view)

Interestingly, for genes Type I/Type I/InsP 5-ptase and E3 Ubiquitin ligase, the respective InDels accounted for an expected segregation pattern among RILs i.e., RILs with a permeability score of 75–100 displayed band pattern similar to *G. max* while RILs with permeability score < 10 depicted band pattern characteristic of *G. soja* (Fig. 5) whereas for another 5 genes, segregation observed among RILs did not reveal any correspondence with the permeability score.

**Fig. 6:**
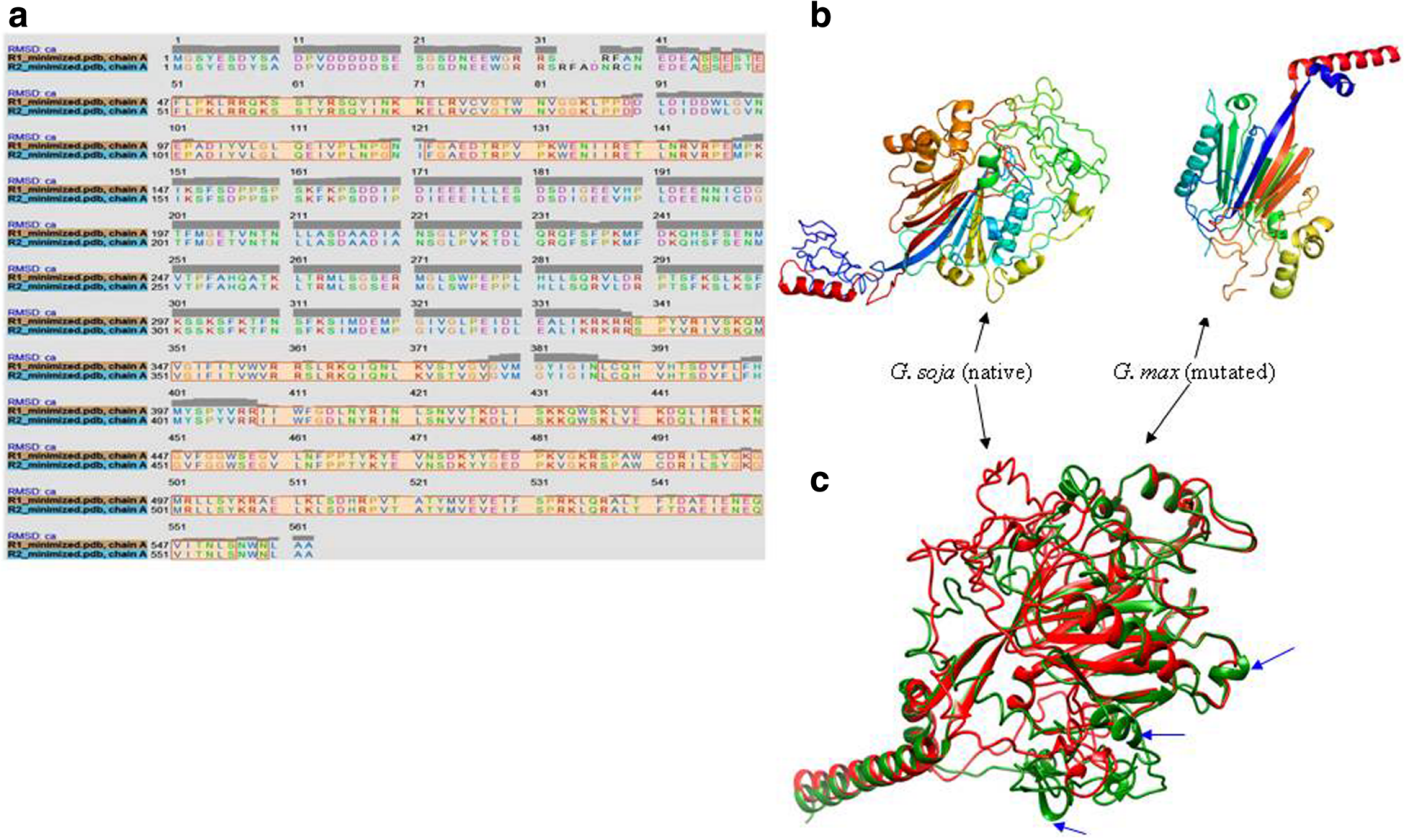
Pairwise alignment (**a**) of TypeI-Inositol polyphosphate5phosphatase1 protein sequence between *G. max* and *G. soja*, (**b**) 3D structure of protein in *G. max* and *G. soja* as predicted by I-TASSER, (**c**) Structural superimposition of protein through Chimera wherein arrows point out the disordered structure of *G. max* (R1 and R2 represent *G. max* and *G. soja*, respectively)

Amino acid sequences of protein Type I/InsP 5-ptase and E3 Ubiquitin ligase of *G. max* and *G. soja* were then used to identify suitable template structures for comparative 3D modeling. A 10 bp insertion in E3 ubiquitin ligase of *G. max*, caused a gain of a stop codon leading to protein truncation, thus structural deviations in its protein structure relative to *G. soja* are obvious and expected (Additional file 11: Figure S5, Additional file 12: Table S2). Of particular interest was the impact of another InDel on the protein conformational structure which was identified as a 12 bp in-frame insertion in *G. soja.* Deletion of 4 amino acids in *G. max* relative to *G. soja* (Fig. 6a) might confer important consequences on protein functioning due to observed conformational distortions and deviations between the two (Fig. 6b). Statistics of the protein modeling with respect to different features are given in Table 3. Selected models for Type I/InsP 5-ptase in *G. max* and *G. soja* displayed accurate topology as governed by the C - score, expected TM-score, RMSD value as well as stabilization of its stereo-chemical properties. Stability of protein structures was further confirmed by Ramachandran plot statistics that revealed the low percentage of amino acid residues to have phi/psi angles in disallowed regions. Further superimposition of refined protein models of *G. max* and *G. soja* resulted in RMSD value of 0.641 Å and revealed major variations in the secondary structure of the protein, i.e., alpha-helix and loop regions that resulted in overall protein conformational changes from its native to the mutated form (Fig. 6c). These changes might be associated with observed and known permeability differences between *G. max* and *G. soja* seeds. Moreover, global alignment of these proteins at the sequence level also confirmed variation at multiple regions. Hence, Type I/InsP 5-ptase was identified as a novel candidate for determination of seed permeability differences among soybean genotypes but needs further validation at protein function level.

**Table 3:** Statistics of TypeI-Inositol polyphosphate5phosphatase1 protein structural predictions in *G. max* and *G. soja* (C score: Confidence score, TM score: Template Modeling score, RMSD: root mean square deviation)

Features		*G. max*	*G. soja*
Top 10 templates predicted by I-TASSER		1i9yA, 1i9yA, 1i9zA, 4cmlA, 4cmnA, 1i9yA, 4cmlA, 4cmnA, 1i9yA, 1i9zA	1i9yA, 1i9yA, 1i9zA, 4cmnA, 4cmnA, 1i9yA, 4cmlA, 2xswA, 1i9yA, 1i9zA
Model Evaluation data of predicted structures	C- score	−2.80	−2.51
	Expected TM score	0.39 ± 0.13	0.42 ± 0.14
	Expected RMSD	14.5 ± 3.7	13.8 ± 3.9
	Number of Decoys	354	455
	Cluster Identity	0.0158	0.0210
Energy value (KJ/mole) of predicted protein models	before energy minimization	− 1148.840	190.541
	after energy minimization	−17,465.256	− 17,325.898
Ramachandran Plot statistics (% residues)	Favored regions	55.9	57.3
	Additional region	31.8	30.8
	Allowed regions	5.9	6.9
	Disallowed regions	6.3	5.1

## Discussion

Whole genome resequencing of different soybean accessions has been reported previously to associate SNPs with numerous traits such as biotic stress, domestication, seed composition, seed size, seed coat, flowering etc. [25]. The present study centered on whole-genome resequencing of *G. max* and *G. soja* to identify variation (SNPs and InDels) present in genes related to seed coat properties with implications on water permeability.

The number of SNPs and InDels identified in the current study are less than those reported earlier due to the high stringency filters used to rule out the possibility of detection of false positive variants and could also be atributed to depth of sequencing. Among soybean cultivars, insertions and deletions of length 1 to 65 bp and 1 to 37 bp, respectively [26] to as high as 500 bp [27] has been reported. The observed average density, i.e., the number of SNPs/Mb is identified to be less while InDels/Mb are observed to be more frequent than those in earlier reports [26]. Mapping of maximum SNPs and InDels on chromosome 18 of soybean cultivars with contrasting *mungbean yellow mosaic India virus* resistance traits has been earlier reported [26] and is consistent with variation distribution observed for *G. max* in the present study. Further, SNPs identified were found to be under the influence of transition biases as expected [28, 29] with Ts/Tv ratios comparable to previous studies [26] indicating the correctness of our workflow. A/G and C/T transitions have also been reported as the most common pattern of nucleotide substitution in white clover [30] and chickpea [31]. A higher frequency of A/G transition was also evident in Desi and Kabuli chickpea [32]. Higher occurrence of the C/T transitions is most likely reasoned to occur due to 5-methylcytosine deamination at CpG dinucleotides over time [33, 34]. Further, codon degeneracy for an amino acid is known to be highest at position three followed by position one and position two, which is clearly reflected from the results obtained that allowed a number of variations to occur at degenerate positions of the codon [35]. More abundant distribution of variants in URR and DRR regions of genes is in agreement with reduced selection pressure and low sequence conservation experienced by regulatory regions. Further, mapping of *G. soja* genes identified a diverse array of pathways and generated a valuable functional data resource in the form of SNPs and InDels, which could be associated with different traits.

**Fig. 7:**
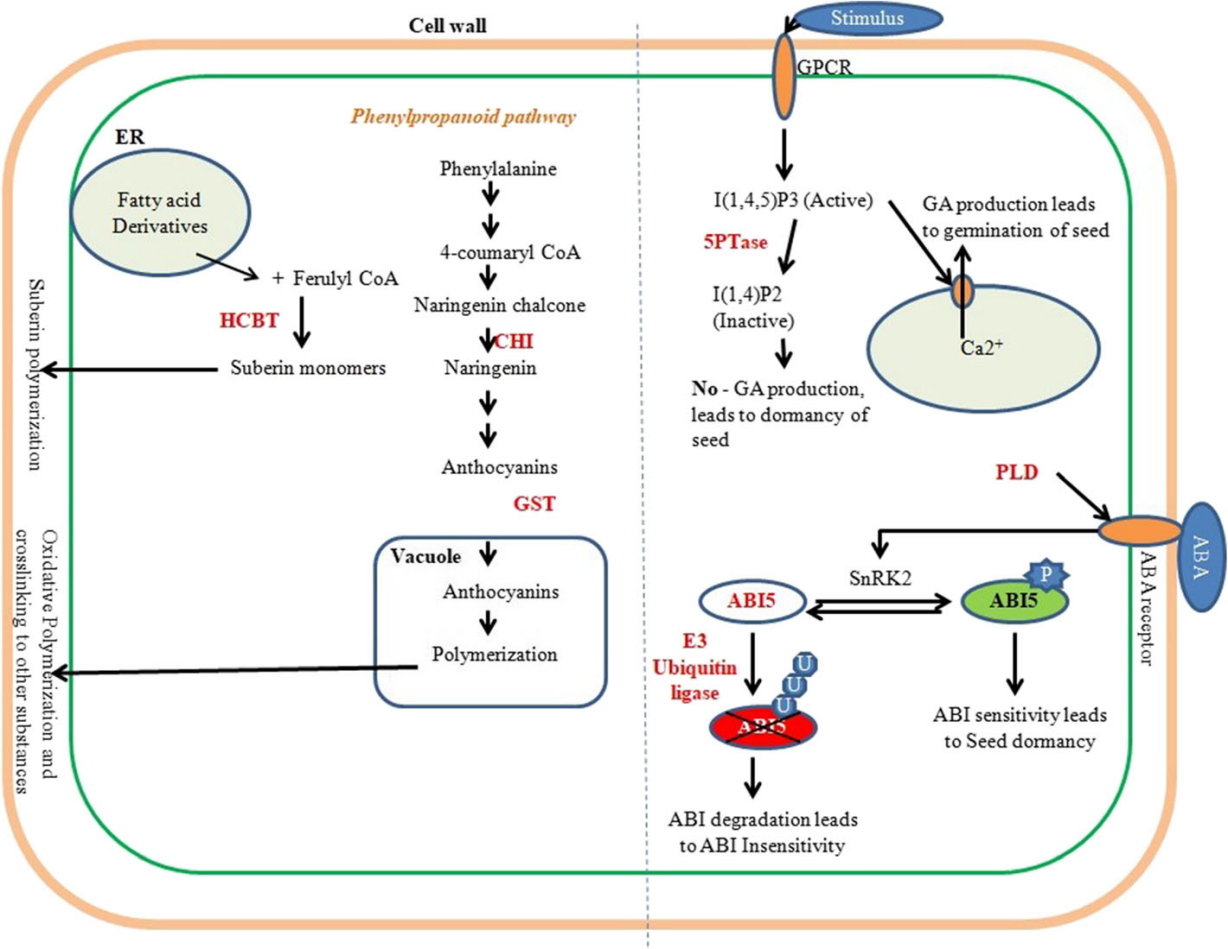
Hypothetical model depicting the role of action of seven selected genes in determining seed coat permeability properties (HCBT: Anthranilate n-hydroxy cinnamoyl/benzoyl transferase, CHI: Chalcone flavone isomerase, GST: UDP:glucose:flavonoid3-O-glucosyltransferases, InsP 5-ptase: TypeI- Inositol polyphosphate 5 phosphatase1, PLD: Phospholipase D and ABI5: Abscisic acid insensitive 5)

Out of this diverse spectrum, factors that could influence seed coat permeability were sorted on the basis of literature as described briefly below and putative role of seven selected genes in governing seed permeability is shown in Fig. 7. Flavonols, Anthocyanins, and Proanthocyanidins are known to be major pigments of testa and correlation between seed coat pigmentation and its water imbibing ability has already been demonstrated in common bean [36], faba bean [37], yardlong bean [38] and guar [39]. Additionally, differentially expressed genes (DEGs) identified between seed coat transcriptome of domesticated pea and its wild progenitor revealed that a majority of them function in the phenylpropanoid pathway followed by flavonoid biosynthetic pathways [40]. Further, glycosylation of flavonoids by UGTs (UDP: glucose: flavonoid3-O-glucosyltransferases) is crucial as its Arabidopsis mutants display reduced seed suberization, cuticle formation and show defect in cellulose biosynthesis, and overall affecting seed permeability [41]. Similarly, BAHD acyltransferases mutant exhibits defective cuticle [42] while GPAT5 acyltransferases mutants are characterized with a reduced amount of suberin aliphatic monomers in Arabidopsis seeds, thereby increasing its permeability [12]. Further, ABI5 is known to negatively regulate seed germination [43–45] while PLD (Phospholipase D) is involved in the expression of the *GAmyb* and *α*-*amylase* gene in aleurone layer of barley seeds [46]. Moreover, CER9 [47] and PROTEOLYSIS6 [48] genes of Arabidopsis encode for E3 ubiquitin ligase and functions in cuticle biosynthesis as well as negatively regulates ABA signaling. Another important component of ABA signaling is InsP 5-ptase, whose mutants exhibit increased ABA sensitivity and faster seed germination. This thus justified the selection of 7 genes that were studied further for their role in governing seed permeability in soybean.

Non-synonymous variation can have several functional impacts due to an altered amino acid sequence that could be manifested in several ways, for example, hampering of interaction between proteins. Thus, to assess their role in influencing gene expression, the allele-specific differential binding effect of non-synonymous variant i.e. binding of a particular motif to a specific allele but not to other was analyzed. Ten TF motifs identified to be present uniquely in *G. soja* laid implications of these onto seed permeability, as reported in previous studies, for example, AP2 is linked to seed dormancy, germination, and longevity in Arabidopsis [49] Similarly, drought responsive Myb transcription factors are associated with the cuticle biosynthesis in legumes [50], which is an important determinant of permeability factor and higher expression of Myb/SANT domain-containing protein i.e. AtSM34, was correlated with Arabidopsis seed germination [51]. Homeobox transcription factors are also important determinants of seed longevity [52]. Thus, with currently available analytical tools, further analysis of these transcription factors in association with seed coat permeability is crucial to decipher the underlying mechanism. Downstream interacting partners of 7 selected genes identified through the variant interaction network and their functional annotation, further specified their role in several seed specific processes, particularly permeability. A similar approach has been used to associate 28 deleterious SNPs identified at a genomic scale with genes involved in plant-pathogen interaction and plant hormone pathways for trait targeting in tomato [53].

Non-synonymous variation can make an impact on the gene expression due to the functional consequences of differential motif binding at variation sites [53]. Gene expression studies of selected genes further supported the hypothesis of their direct or indirect involvement in controlling soybean seed permeability. Transcript abundance of CHI gene at different hours of imbibition in *G. max* and *G. soja* seeds correlated well with its role in the production of flavonoids to regulate water movement across the seed coat. Similarly, genes involved in phenyl proponoid pathway and glycosyl transferases exhibit 2 fold up-regulation in the seed coat of black soybean in comparison to the brown seed coat [54]. Upregulation of UDP-glucosyl transferase expression in cultivated pea relative to its wild progenitor [40] is in coherence with the current study. More abundant ABI5 expression in dormant sorghum cultivars [55] as well as in wild, desiccating and dry seeds of Arabidopsis relative to its vegetative tissues has been reported [56]. Similar to the current findings, TaABF, an orthologue of ABI5 in wheat undergoes a reduction in transcript abundance during imbibition of non-dormant seeds in comparison to the transient increase experienced by dormant grains [57]. Lastly, PLD inhibitors are reported to induce the altered emergence of radicle and cotyledons causing inhibition of seed germination [58, 59].

Further, marker development from SNPs/InDels identified from sequencing data has been done successfully for various agronomical important traits. InDels identified in gene Type 1/InsP5-ptase (12 bp) and E3 Ubiquitin ligase (10 bp) in the current study also revealed segregation in a RIL population in correlation with the permeability scores of a particular RIL and the banding pattern characteristic for *G. max* or *G. soja.* This revealed a preliminary, but a relevant association of these InDels with observed seed permeability differences between cultivated soybean and its wild progenitor and thus enhanced the significance of the present study that could be used to develop simple and efficient PCR and gel-based molecular markers. In a previously reported study, 22 InDel markers (56-432 bp) were developed in rice to reliably distinguish all genome types of the genus Oryza and were found useful for maintenance of germplasm stocks [60], 2 InDel markers to distinguish rice varieties with *Lgc1* gene [61] and yield-related functional genes [62] has been reported. Comparative analysis of 4 accessions of chickpea revealed 21,499 genome-wide InDels of length 2-54 bp and identified 5 InDel marker containing candidate genes linked to flowering and maturity time QTLs [63]. In tomato, 2272 polymorphic InDels (1-94 bp) were evaluated in 22 tomato lines to assist in gene cloning and marker-assisted selection [64]. In soybean, InDel markers were used to fine map crinkly leaf locus to a 360 Kb region on chromosome 7 [65]. Further, Genome-wide analysis of 106 soybean genomes representing the wild, landraces, and elite lines was conducted to discover variation and to associate this variation (SNPs and InDels) with agronomical important and domestication-related traits and for identification of novel alleles [66]. This demonstrates the practical utility of developing InDel markers to expedite genomics-assisted breeding applications.

InDels could have more impact on protein structure and function than single base changes [67], thus allowing their use for development of phylogenetic markers. The evolutionary patterns of InDels from 35 eukaryotic proteomes have recently been studied, including model crop plants, i.e., Arabidopsis and rice [68]. Additionally, protein conformational changes due to InDels resulting in major trait differences in mitochondrial genes are well known. Further, InDels can have a more intense impact on the overall protein structure if they occur within α-helices and ß-sheets while loops and turns are known to accommodate InDels comparatively well [69]. The observed protein conformational differences at the secondary level for InsP5-ptase due to deletion present at DNA level in *G. max* relative to *G. soja* thus might be responsible for their observed differences in seed permeability. Thus, InDels especially those altering protein structures and function have the potential to improve our understanding of the consequence of observed natural variation in a better way.

## Conclusion

The present study is a comprehensive report on the comparison of the genomes of *Glycine max* and its wild progenitor (*G. soja*) to decipher and annotate the potential genic variation in an effort to understand seed permeability as evidenced by transcript abundance of genes and water permeability scores. In summary, two candidate genes were identified that hold the potential to be associated with differentiating permeability abilities of soybean seeds. Elucidated genomic information, thus provides a valuable resource to facilitate trait dissection based on detection of sequence-based variation with implications for molecular breeding.

## Methods

### Wet lab analysis

#### Plant material and DNA isolation

Soybean genotypes with contrasting seed permeability traits were selected, i.e., *G. soja* accession DC2008–1 (hard seed) and *G. max* accession DS9712 (soft seed). Plant material (Leaf and seed samples) was procured from Division of Genetics, IARI, Pusa Campus, New Delhi. Genomic DNA was isolated from leaves of parental genotypes and300RILs (F9) derived from their cross using the standard CTAB method [70] and DNAsure Plant Mini Kit (Nucleo-pore). 18 RILs (F8 generation) were randomly selected on the basis of water permeability scores (unpublished data) i.e., 9 RILs with a score in the range of 0–10 and other 9 RILs with a score in the range of 75–100 for further analysis. For permeability tests, 20 seeds each of 160 RILs, *G. soja* accession DC2008–1 and *G. max* accession DS9712, in triplicates were soaked in 100 ml of distilled water for 6 h at room temperature (25 °C) as per modified Zhang et al. [21]. After 6 h, seeds were checked for imbibition. The seeds that had not imbibed water were counted as “impermeable or hard seeds” while the seeds that had imbibed water were counted as “permeable or soft seeds”. Seed coat permeability was defined as the percentage of soft seeds that had imbibed water after soaking for 6 h. Additional file 13: Figure S6 depict the seeds of *G. soja* and *G. max* before and after imbibition.

#### Whole genome resequencing

Genomic DNA from 25 days-old seedlings of *G. soja* and *G. max* was isolated using the DNAsure Plant Mini Kit (Nucleo-pore) and its quality and quantity were ascertained by Bioanalyzer 2100 (Agilent Technologies). DNA library was prepared using TruSeq DNA PCR-Free HT Sample Preparation Kit, following the manufacturer’s instructions and was processed for paired-end sequencing using the Illumina HiSeq 1000 platform (Illumina Technologies).

#### Primer designing and DNA polymorphism studies of candidate genes

Primer pairs targeting the variation in 7 selected genes were designed using an IDT PrimerQuest tool (https://eu.idtdna.com/PrimerQuest/Home/Index) with amplicon size ≤500 bp. Primer pairs were custom synthesized through the facility of Eurofins Analytical Services, India Private Ltd. and were validated using genomic DNA of *G. max and G. soja*. PCR was performed in 20-μl volume in Takara Thermocycler. Reaction mixture contained 0.5X buffer, 5 μM forward + reverse primer, 1.5 mM MgCl_2_, 0.8 mM dNTPs, 1 unit of Taq DNA polymerases (New England Biolabs) and 100 ng of genomic DNA. PCR amplification conditions were: 95 °C for 5 min, followed by 40 cycles of 95 °C for 30 Sec, Tm (°C) for 30 Sec and 72 °C for 30 Sec and a final step of extension at 72 °C for 5 min. After amplification, 2 μl of 6X loading dye was added to each of the amplified products which were then resolved in 4% Metaphor-Agarose gels (TBE buffer) supplemented with ethidium bromide. Sequences of primer pairs designed are listed in Additional file 14: Table S3.

#### RNA isolation and reverse transcription quantitative PCR (RT-qPCR) analysis

RNA extraction was performed on 2 replicates of 5 seeds of *G. max* and *G. soja* each at 5 different time intervals, i.e., 0, 12, 24, 48 and 72 h of imbibition using RaFlex™ total RNA Isolation kit (Merck Millipore) as per manufacturer’s protocol. RNA quantity and purity was ascertained using NANODROP 2000 (Thermo Scientific) and formamide denaturing gel electrophoresis.For RT-qPCR, 1 μg of total RNA was reverse-transcribed using RevertAid First strand cDNA synthesis kit (Thermo Scientific) according to the manufacturer’s instructions. First strand cDNA was further diluted to 1:10 ratio for real-time expression studies performed on Roche Light Cycler 480 using SyBr GreenER qPCR Supermix (Invitrogen). Gene expressions from each cDNA sample were normalized to the endogenous reference gene i.e., 18SrRNA. Forward and reverse primers used for RT-qPCR analysis are listed in Additional file 14: Table S3. Relative expression levels were calculated using the comparative 2Δ(Ct) method [71] and standard errors were calculated.

### Bioinformatics analysis

#### Read mapping and discovery of SNPs and InDels

Initial quality control and cleaning of reads were done using FastQC (http://www.bioinformatics.babraham.ac.uk/projects/fastqc/) Trimmomatic software at default parameters. High-quality Illumina reads of wild and cultivated soybean were mapped to the reference genome of *G. max* (var. Williams 82, ftp://ftp.ensemblgenomes.org/pub/plants/release-35/fasta/glycine_max/dna) utilizing Burrows-Wheeler Aligner (BWA) software (v0.7.10). Samtools and Picard (http://broadinstitute.github.io/picard/) were used to refine the mapping output of BWA, which was further filtered at mapping quality (MQ ≥ 20) and base quality (Q ≥ 30) for downstream analysis. Genome-wide SNPs and InDels calling was done using Genome Analysis Toolkit (GATK, v3.1.1) software (https://software.broadinstitute.org/gatk/) and Samtools/bcftools (https://samtools.github.io/bcftools/) at a minimum read depth of 10 and SNP quality 30. The Realigner Target Creator and InDels-Realinger features of GATK were used to realign InDels. To further rule out identification of false positives, following criteria were used: (cluster size 3, cluster window size 10, and filter DP < 10, Filter MQ 0 > = 4 (MQ 0 / (1.0* DP) > 0.1).

#### Chromosomal distribution of SNPs

Genomic distribution and annotation of the SNP and InDels were analyzed using in-house Perl scripts and visualized with Circos software (http://circos.ca/software/). To assess their distribution, variant positions were integrated with a GFF3 file containing annotation data of *G. max* (ftp://ftp.ensemblgenomes.org/pub/plants/release-35/gff3/glycine_max/).

#### Functional annotation and categorization of SNPs and InDels

Genes carrying variation were annotated into KEGG pathways with KeggMapper (http://www.genome.jp/kegg/mapper.html) using *G. max as* a reference. Synonymous/ Non-synonymous SNPs and impact of variation in gene function were evaluated using Variant effect Predictor software (http://www.ensembl.org/info/docs/tools/vep/index.html) in reference to *G. soja*. Further, SIFT22 (subPSEC score ≤ − 3) was used to detect deleterious SNPs.

#### Presumptive effect of deleterious SNPs/ InDels at structure level and interacting network

Impact of SNPs and InDels in selected genes was analyzed at the sequence level by prediction of differential binding events utilizing 8 mer motif model and 10 bp flanking gene sequences w.r.t. identified variation via CIS-BP web server (http://cisbp.ccbr.utoronto.ca/TFTools.php). Further, high confidence interacting partners of selected genes were identified using SoyNet (http://www.inetbio.org/soynet/), which gene to gene relationship based on Bayesian statistics to identify candidate genes for the hypothesis in consideration and interaction network was build and visualized using Cytoscape [72]. Gene ontology (GO) analysis of interacting partners was conducted using an AgriGO program (http://bioinfo.cau.edu.cn/agriGO/).

#### 3-D structure prediction, validation, visualization and analysis

The three-dimensional structure of variant Type I- Inositol polyphosphate 5 phosphatase1 genes and E3 Ubiquitin ligase from *G. max* and *G. soja* were predicted by I-TASSER (http://zhanglab.ccmb.med.umich.edu/I-TASSER/). Predicted protein models were subjected to Structural Analysis and Verification Server (SAVES) (http://services.mbi.ucla.edu/SAVES) for evaluation and quality checking. Refined protein models were visualized and superimposed by Chimera1.11 (https://www.cgl.ucsf.edu/chimera/).

## Additional files

Additional file 1: **Table S1.** Statistics of Assembly generated from Illumina HiSeq1000 sequencing data. (DOC 29 kb)

Additional file 2: Identification of total SNPs and InDels in *G. max* with respect to the reference genome. (XLSX 44647 kb)

Additional file 3: Identification of total SNPs in *G. soja* with respect to reference genome. (XLSX 19576 kb)

Additional file 4: Identification of total InDels in *G. soja* with respect to reference genome. (CSV 97568 kb)

Additional file 5: Functional classification of non-synonymous SNPs and InDels into diverse pathways. (XLS 636 kb)

Additional file 6: **Figure S1.** GO annotation of 205 interacting partners of 7 selected genes into biological processes. (PNG 309 kb)

Additional file 7: **Figure S2.** GO annotation of 205 interacting partners of 7 selected genes into molecular functions. (PNG 307 kb)

Additional file 8: **Figure S3.** GO annotation of 205 interacting partners of 7 selected genes into cellular compartments. (PNG 31 kb)

Additional file 9: Functional annotation of 205 interacting patterns as identified for 7 selected genes. (XLS 184 kb)

Additional file 10: **Figure S4.** DNA polymorphism analysis of genes in *G. max* (Gm) and *G. soja* (Gs) in order Anthranilate n-hydroxy cinnamoyl/benzoyl transferase, Chalcone flavone isomerase, Abscisic acid insensitive 5, Phospholipase D, TypeI- Inositol polyphosphate 5 phosphatase1, E3 Ubiquitin ligase and Glycosyltransferase. (JPG 111 kb)

Additional file 11: **Figure S5.** Pairwise alignment (a) of protein sequence of E3 Ubiquitin ligase from *G. max* and *G. soja*, (b) 3D structure of protein in *G. max* and *G. soja* as predicted by I-TASSER, (c) Structural superimposition of protein obtained through Chimera (Gm and Gs represent *G. max* and *G. soja*, respectively). (JPG 154 kb)

Additional file 12: **Table S2.** Statistics of E3 Ubiquitin ligase protein structural predictions in *G. max* and *G. soja* (TM score: Template Modeling score, RMSD: root mean square deviation). (DOC 34 kb)

Additional file 13: **Figure S6.** Seeds of *G. soja* and *G. max* before (Control seeds, 0 h) and after water imbibition of 6 h. (JPG 7 kb)

Additional file 14: **Table S3.** List of forward and reverse primers designed for detection of DNA polymorphism and real-time analysis for selected genes. (DOC 30 kb)

## Abbreviations

ABA: Abscisic acid
ABI: Abscisic acid insensitive
InDels: Insertion deletions
RFLP: Restriction fragment length polymorphism
RIL: Recombinant inbred line
SNP: Single nucleotide polymorphism
SSR: Simple sequence repeat
