# Supplementary material for: Genome-wide identification and characterization of InDels and SNPs in Glycine max and Glycine soja for contrasting seed permeability traits
Source: BMC Plant Biol. 2018 Jul 9;18:141. doi: 10.1186/s12870-018-1341-2 (PMC6038289; doi:10.1186/s12870-018-1341-2)
Supplement: Supplementary file 1 — Table S1. Statistics of Assembly generated from Illumina HiSeq1000 sequencing data. (DOC 29 kb) [file 12870_2018_1341_MOESM1_ESM.doc]

**Table S1:** Statistics of Assembly generated from Illumina HiSeq1000 sequencing data

| **Mapping statistics** | ***G. max*** | ***G. soja*** |
| --- | --- | --- |
| **Total reads** | 118805500 | 119804116 |
| **Mapped reads** | 117110216 (98.57%) | 113668324 (94.87%) |
| **Unmapped reads** | 1716554 (1.44%) | 5934678 (4.95%) |
| **Properly paired** | 107297453 (90.31%) | 98475387 (82.19%) |
| **Singletons** | 9458731 (7.96%) | 15804556 (13.19%) |
| **Coverage** | 12.56 | 12.32 |
