# Supplementary figures and images for: Genome-wide identification and characterization of InDels and SNPs in Glycine max and Glycine soja for contrasting seed permeability traits
Source: BMC Plant Biol. 2018 Jul 9;18:141. doi: 10.1186/s12870-018-1341-2 (PMC6038289; doi:10.1186/s12870-018-1341-2)

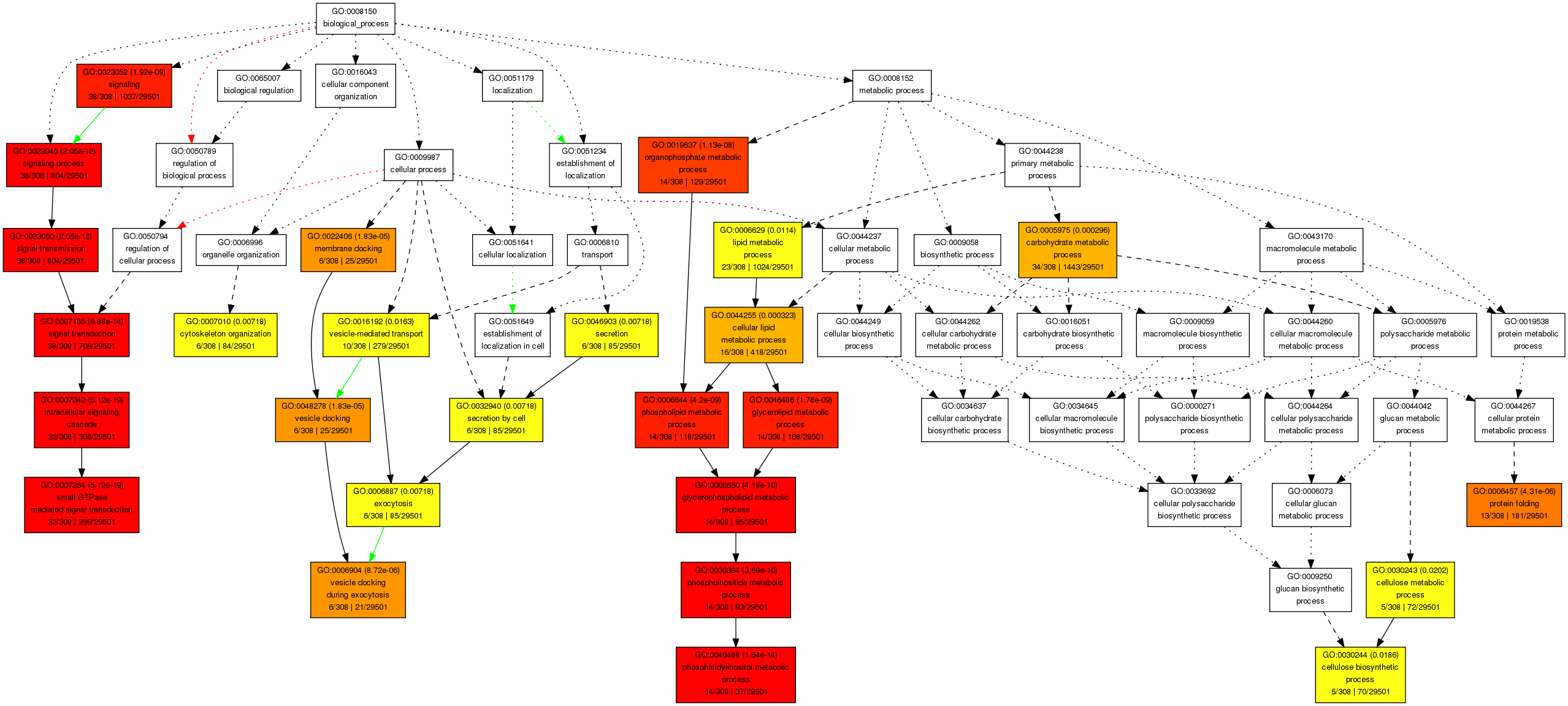

Supplement: Supplementary file 6 — Figure S1. GO annotation of 205 interacting partners of 7 selected genes into biological processes. (PNG 309 kb) [file 12870_2018_1341_MOESM6_ESM.png]

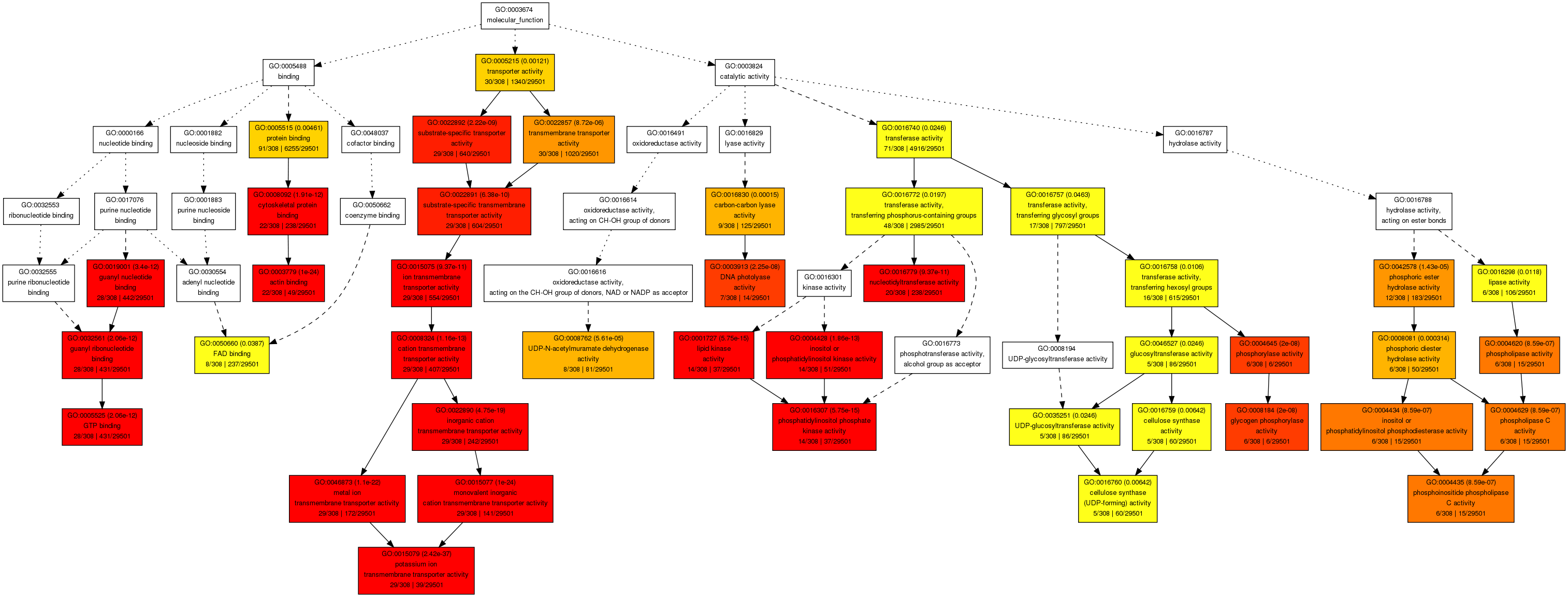

Supplement: Supplementary file 7 — Figure S2. GO annotation of 205 interacting partners of 7 selected genes into molecular functions. (PNG 307 kb) [file 12870_2018_1341_MOESM7_ESM.png]

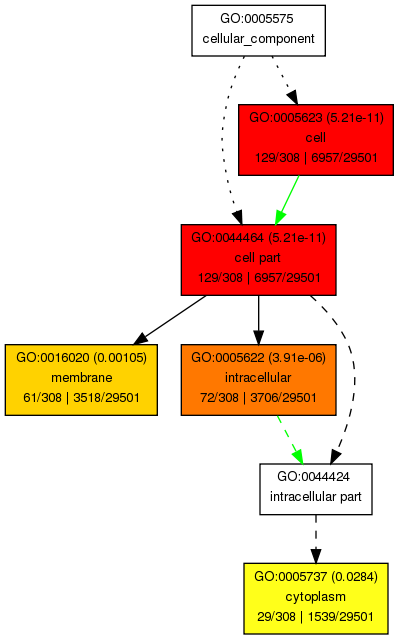

Supplement: Supplementary file 8 — Figure S3. GO annotation of 205 interacting partners of 7 selected genes into cellular compartments. (PNG 31 kb) [file 12870_2018_1341_MOESM8_ESM.png]

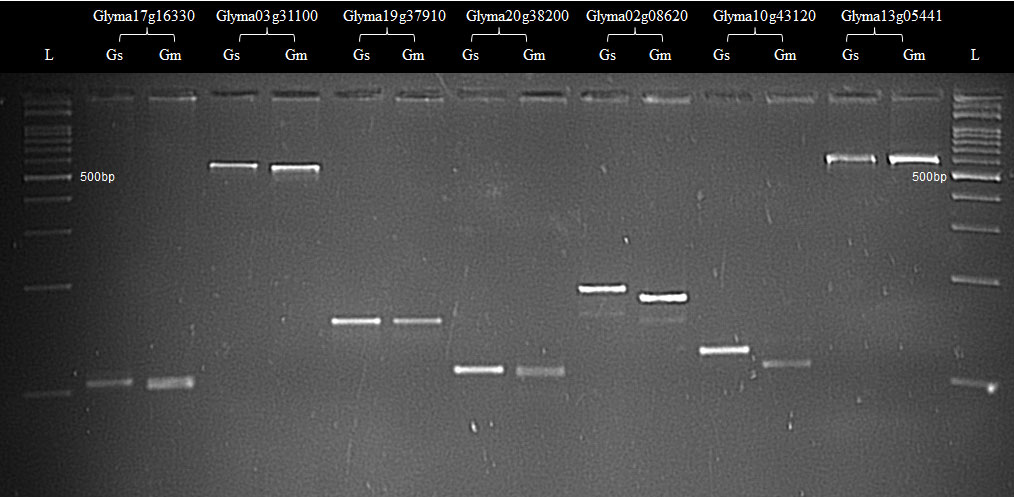

Supplement: Supplementary file 10 — Figure S4. DNA polymorphism analysis of genes in G. max (Gm) and G. soja (Gs) in order Anthranilate n-hydroxy cinnamoyl/benzoyl transferase, Chalcone flavone isomerase, Abscisic acid insensitive 5, Phospholipase D, TypeI- Inositol polyphosphate 5 phosphatase1, E3 Ubiquitin ligase and Glycosyltransferase. (JPG 111 kb) [file 12870_2018_1341_MOESM10_ESM.jpg]

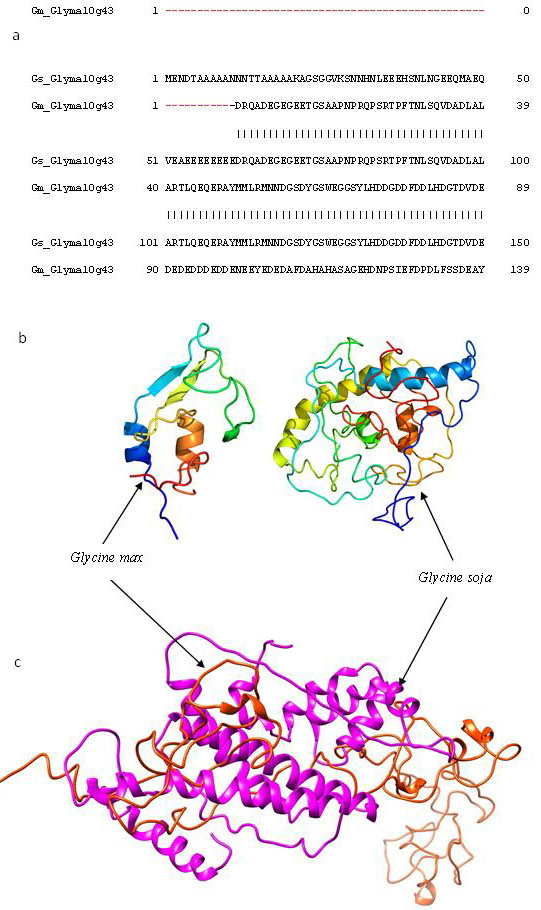

Supplement: Supplementary file 11 — Figure S5. Pairwise alignment (a) of protein sequence of E3 Ubiquitin ligase from G. max and G. soja, (b) 3D structure of protein in G. max and G. soja as predicted by I-TASSER, (c) Structural superimposition of protein obtained through Chimera (Gm and Gs represent G. max and G. soja, respectively). (JPG 154 kb) [file 12870_2018_1341_MOESM11_ESM.jpg]

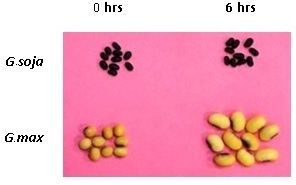

Supplement: Supplementary file 13 — Figure S6. Seeds of G. soja and G. max before (Control seeds, 0 h) and after water imbibition of 6 h. (JPG 7 kb) [file 12870_2018_1341_MOESM13_ESM.jpg]
