# Supplementary material for: Genome-wide identification and characterization of InDels and SNPs in Glycine max and Glycine soja for contrasting seed permeability traits
Source: BMC Plant Biol. 2018 Jul 9;18:141. doi: 10.1186/s12870-018-1341-2 (PMC6038289; doi:10.1186/s12870-018-1341-2)
Supplement: Supplementary file 12 — Table S2. Statistics of E3 Ubiquitin ligase protein structural predictions in G. max and G. soja (TM score: Template Modeling score, RMSD: root mean square deviation). (DOC 34 kb) [file 12870_2018_1341_MOESM12_ESM.doc]

**Table S2:** Statistics of E3 Ubiquitin ligases protein structural predictions in *G. max* and *G. soja* (TM score: Template Modeling score, RMSD: root mean square deviation)

| Features | | *G. max* | *G. soja* |
| --- | --- | --- | --- |
| Top 10 templates predicted by I-TASSER | | 5ijoJ, 4v3lC, 4v3lC, 4ke2A, 2l0bA, 5gaoE, 4gwpB, 2l0bA, 5d0iA, 3j65R | 2nbiA, 3k1lA, 5dfzC, 4v3lC, 5gaoE, 4v3lC, 5ijnF, 4v3lC, 1kvpA, 2l0bA |
| Model Evaluation data of predicted structures | C- score | -3.18 | -1.05 |
|  | Expected TM score | 0.36±0.12 | 0.58±0.14 |
|  | Expected RMSD | 13.4±4.1Å | 8.9±4.6Å |
|  | Number of Decoys | 1052 | 600 |
|  | Cluster Identity | 0.0418 | 0.25 |
| Energy value (KJ/mole) of predicted protein models | before energy minimization | -2514.1 | 634.121 |
|  | after energy minimization | -19285 | -12565 |
| Ramachandran Plot statistics (% residues) | Favored regions | 67.4 | 67.8 |
|  | Additional region | 42.8 | 41.8 |
|  | Allowed regions | 6.8 | 7.1 |
|  | Disallowed regions | 7.8 | 7 |
