# Supplementary material for: Genome-wide identification and characterization of InDels and SNPs in Glycine max and Glycine soja for contrasting seed permeability traits
Source: BMC Plant Biol. 2018 Jul 9;18:141. doi: 10.1186/s12870-018-1341-2 (PMC6038289; doi:10.1186/s12870-018-1341-2)
Supplement: Supplementary file 14 — Table S3. List of forward and reverse primers designed for detection of DNA polymorphism and real-time analysis for selected genes. (DOC 30 kb) [file 12870_2018_1341_MOESM14_ESM.doc]

**Table S3:** List of forward and reverse primers designed for detection of DNA polymorphism and real time analysis for selected genes

| **Gene (Glyma Ids)** | **PCR primer sequences** | | **qRT-PCR primer sequences** | |
| --- | --- | --- | --- | --- |
|  | **Forward primer** | **Reverse primer** | **Forward primer** | **Reverse primer** |
| Glyma17g16330 | TCGATTTAACTCCCTGGGATCTTC | TTGGAGGTGCTCGATTTGGTTT | TTGAAGTGTGCCTTCCTTATGT | TAGTTGGAAATGGCATCCATGA |
| Glyma03g31100 | GTTTCCACTTGGTAAAGCCAAAG | TACCTTCTTGCGATGTCTTCTG | GAAGTGGGAAGCATCCAGAGTAA | GCTCAATTTCTTCTTTGGCCTGAT |
| Glyma19g37910 | CCGCATTACGCGAATAATAACAAC | CACCATTATGATCCCTCTTACCATAC | GAGGATGATCAAGAATAGAGAGTCAG | TGTTTCAGTTGGGAGTTCTCTT |
| Glyma20g38200 | GCTGATGGACTAGATACTTCAAATGG | TTCATCTGGGTGTCTGAAACTAAAG | GGTCTGTCATGAGAATGGAGAAA | GCTCTCAATAACAGCTGGTCTAA |
| Glyma02g08620 | GCCAGTCTTGAATGCGTGT | CTCACTTTGTGTTTGGTGTGC | - | - |
| Glyma10g43120 | CTGTTACTTCTAACCTTAACTGTGTCC | AAGGACTCACCATCCTCATAGTC | GCAGAGGTCTTTCAACTGATACTATTG | GGTTAGAAGTAACAATCTGCACATACG |
| Glyma13g05441 | GCTGGATAGGACCTTCGATTATAG | TACCCAATAGCCAGTAGCAATATC | GCTTTGCTGCCAGTGTATCT | GAATGAACCTGGTGTGCTATGT |
